# Lectures on Clinical Medicine—Diseases of the Heart

**Published:** 1845-07

**Authors:** 


I. Lectures on Subjects connected with Clinical Medicine, comprising Diseases of the Heart. By *P. M. Latham*, M.D., Physician Extraordinary to the Queen, and late Physician to St. Bartholomew's Hospital. Vol. I. pp. 374. London, 1845. Longman and Co.

II. The Diagnosis, Prevention, and Treatment of Diseases of the Heart, and of Aneurism, with Observations on Rheumatism. By *J. J. Furnivall*, M.D. late Physician to the General Infirmary at Hertford, &c. &c. 8vo. pp. 216. London, 1845. Churchill.

The present volume will unquestionably add to Dr. Latham's reputation as an acute observer and a vigorous describer of disease. His is not the character of one who loves to follow in the steps of another, and seeks to uphold his own views by appealing to the experience of any who have gone before him. He looks with his own eyes, and writes too with his own pen, in a bold and firm style that admits of no ambiguity, and is legible by all. Our readers may be aware that he published a volume "On Subjects connected with Clinical Medicine," in 1836—a review of which will be found in the number of this Journal for July of that year—that was well received by the profession, and that at once stamped its author as one of the ablest practical writers of the day. His intention was to have followed it up by other volumes on the same subject; but ill-health, we regret to find, and other causes came in the way, and have prevented him from fulfilling it until the present time.

The series of Lectures now before us is devoted almost exclusively to the pathology of the Heart, or rather to the history and treatment of those two most important diseases—the parent source of most others—of this organ, Endocarditis and Pericarditis. Our author prefaces his description of them with some excellent observations on the Auscultation of the Heart, in health and in disease. The first four lectures contain a capital summary of all that is necessary or really useful to know on this branch of diagnosis, and we can with confidence recommend them as forming one of the best text-books for the guidance of men engaged in general practice.

As a matter of course, it is only to such passages as contain something new, or which place what is already known in a somewhat novel light, that we shall at present direct the reader's attention.

The normal situation of the heart's impulse is usually described to be "between the cartilages of the 5th and 6th left ribs, at a point about two inches below the nipple, and an inch on its sternal side." (Hope). But the exact spot, it should be remembered, varies somewhat in the same person, according to the position in which the patient is placed.

"The same man, according to the varying postures of his body, will alter the place and extent of this impulse. He stands up, and makes it felt just where the apex strikes the chest, at a point between the fifth and sixth ribs, and not beyond it. He leans forward, and makes it felt both at this point and a little above it, and in the direction of the sternum. He reclines upon his back, and renders it almost or altogether imperceptible anywhere. He turns on his left side, and renders it more perceptible than ever, and in a somewhat larger and different space, between the fifth and sixth ribs, and from thence more towards the mamma than the sternum. Again, he turns on his right side, and again he renders the impulse almost or altogether imperceptible." 14.

The *præcordial* (as Dr. Latham calls it), or—to prevent misconception, from this term having hitherto been generally used in a somewhat different sense—the *Cardiac, region* in the healthy state may be tolerably well defined by describing a circle, two inches in diameter, from a point in the cartilage of the fifth left rib, midway between its sternal and costal ends. "In the space indicated, most practical men would (I think) be ready to admit that percussion conveys to the ear a sense rather of less resonance than of positive dulness. The fact is, if the percussion used be but of moderate force, you must listen attentively to make sure that the resonance is really less here than elsewhere. It is only when the percussion used is of a force somewhat painful to the patient, that the ear begins to acknowledge a positive dulness."

It should be remembered that the dulness of the sound thus elicited is always more distinct in the erect than in the reclining position, and at the time of expiration than that of inspiration.

No part of Dr. Latham's description pleases us better than his account of the abnormal sounds, which so frequently accompany the movements of the heart. Let us briefly notice it. In the first place, he recommends that all these irregular sounds should be called *murmurs*, to distinguish them from the healthy sounds or natural tictac of the organ; and, inasmuch as they are always produced by certain morbid states, either of the cavities of the heart or of its outer surface, he proposes to divide them into such as are *endocardial* and those which are *exocardial*. To the first belong all the murmurs that accompany either the first or the second cardiac sounds, and which are usually described as being of a blowing, sawing, filing, or rasping character. The second includes all the rubbing, rustling, or crumpling noises or bruits, which have been noticed by different writers in pericardial inflammation, whether this be of an acute or of a chronic character.

The one set accompanies or, it may be, disguises and supersedes the natural sounds of the heart; the other set is superadded to, and is quite independent of, them. The former always seem to the ear to be more distant or deeply-seated than the latter; they are usually, also, shorter in their duration. The *exocardial* murmurs are not so fluctuating and liable to change within a brief space of time as the *endocardial;* they may indeed change from one day to another; but rarely, if ever, from hour to hour, as may happen in the case of the latter.

We need scarcely say that persistent Endocardial murmurs are almost always indicative of a morbid state of the valves—generally either the mitral or the aortic; for the valves on the right side of the heart are very rarely diseased. Beyond this amount of information, our diagnosis must remain in most instances obscure and uncertain; except indeed when the murmur is invariably synchronous with the *diastole;* for then it very generally proceeds from the aorta, and is a sound of regurgitation. When the murmur is *systolic,* it may come either from the aortic or from the mitral valve: in the latter case, it is caused by the reflux of blood from the ventricle into the auricle. Some degree of assistance to our diagnosis may sometimes be obtained by attending to the *direction,* in which the valvular murmur seems to be prolonged from the point whence it proceeds: but this can often not be done with any degree of certainty. After admitting that it is scarcely possible to distinguish a mitral from an aortic murmur by any difference in their primitive seat or locality—all the valves being clustered together in so small a space, that the mouth of a common stethoscope must cover them—our able guide observes:—

"But suppose we raise our ear, or the stethoscope, from this exact spot, and shift it an inch or two higher or an inch or two lower. Higher we may hear the endocardial murmur still, and lower we may lose it altogether. Or higher we may lose it altogether, and lower we may hear it still. Or both higher and lower we may still distinctly hear it. By this procedure we are following the endocardial murmur in the direction it takes after it leaves the orifice from which it is propagated, and we find how various the direction is, upwards in one case, downwards in another, and both upwards and downwards in a third. But still it is the orifice, from which it is propagated, that gives the murmur its particular direction; and this (it is said) may be taken for a general fact.

"Accordingly, when the endocardial murmur is conveyed in an upward direction, even above the basis of the heart, and still along the course of the aorta, and further still, as sometimes happens, along the subclavian and carotid arteries, the aortic orifice is its point of departure, and the valve, there situated, is the valve diseased. When it is conveyed in a downward direction, and to the apex of the heart, the auriculo-ventricular orifice is its point of departure, and the valve, there situated, is the valve diseased. And when it is conveyed both in an upward and downward direction, both in the course of the aorta, and to the apex of the heart, then it has two points of departure, and both the aortic and the mitral valves are diseased." 43.

On this, however, and some other points connected with the diagnosis of valvular disease, Dr. Latham does not hesitate to acknowledge that, at the present moment, "he is less peremptory about the certainty of their application than he was a year or two ago." Such is the uncertainty that still hangs over much of cardiac semeiology. Let not the reader therefore be misled by the confident asseverations of not a few of the auscultatory writers of late years.

It is too generally believed that, in cases of valvular disease, the loudness of the abnormal murmurs is proportionate to, and may be regarded as a test of, the amount of contraction of the cardiac orifice or orifices affected. This opinion must be received with some degree of reservation.

" The truth is, that the murmur becomes louder as the disease and the impediment increase only *up to a certain point*, and then, that it becomes less and less loud as they go on to increase beyond this point. Thus the disease and the impediment still increasing may, and sometimes do, reach a point at which the endocardial murmur ceases thenceforth, and altogether, as long as life remains.

" Two individuals of unsound heart died within a few days of each other. I witnessed the symptoms of their disease during life, and after death I saw what that disease actually was. In both the right ventricle was dilated, and the left was dilated and hypertrophied; and in both the mitral valve and the aortic valve were diseased. But the valvular disease, and the impediment resulting from it, were far greater in one case than in the other. In the one the auriculo-ventricular orifice was so narrowed as only just to admit the little finger, and the aortic orifice was only just not closed. In the other there remained a tolerably free space for the passage of blood through both orifices.

" Now in the first case during life there was no endocardial murmur at all; while during life in the second there was a loud bellows-murmur audible in the whole præcordial region, and far on either side of it, and beyond it upwards in the course of the aorta.

" All this seems to admit of easy explanation. When endocardial murmurs result from diseased valves, there are two agents engaged in producing them, viz. the mechanical obstacle which the blood encounters, and the blood itself. It is from unusual vibrations among the particles of the blood that the unusual sound immediately proceeds; but it is the obstacle which sets the conflict agoing. Now the sound must be in proportion to the vibration; and the vibration is in proportion to the amount of the obstacle and the quantity of blood and the rate at which it circulates taken together. Thus the endocardial murmur becomes louder and louder while the valvular disease is upon the increase, as long as the heart by its increasing thickness is still able to force a large current of blood through a moderately contracted orifice. But the endocardial murmur becomes fainter and fainter, and at length ceases altogether, as the valvular disease, by its further increase, goes on still to narrow the orifice, and the ventricle with all its increasing thickness can only force the blood through it in a more and more slender stream." 50.

Dr. Latham attaches but little importance to the particular *kind* or description of murmur that may chance to be heard in any case of cardiac disease. The catalogue of blowing, filing, sawing, rasping, cooing, &c. sounds is more ingenious than useful. "Upon the whole, my persuasion is," says he, "that no practical good has come from curiously naming, and noting, and multiplying endocardial murmurs. The *mere* murmur can only tell me whether it proceed from the inside or from the outside of the heart. For more than this I cannot trust it. But in telling me this, it tells that which I have no possible means of knowing without it."

Every practical man is aware that certain endocardial murmurs may exist without the presence of any organic disease of the valves or other parts of the Heart. A loud bellows-murmur is a very common phenomenon in various hæmorrhagic, hysterical, and chlorotic maladies. In children, too, it is not an unfrequent occurrence, whenever the force of the circulation is much increased. Any impediment also to the free action of the heart, although the organ itself is perfectly sound in structure, is apt to give rise to various endocardial murmurs. This is often the case in cases of deformity of the chest, when the heart is either displaced, or hemmed in and compressed. If the patient be young, and the ribs yielding, the mere pressure of the end of the stethoscope, pretty firmly applied, will sometimes suffice to induce a bellows-murmur, which is not perceptible when the pressure is removed. It is therefore necessary, under such circumstances, to be on our guard, lest, by our own awkwardness, we aggravate, if we do not actually cause, a sound that may be supposed to be symptomatic of disease.

"The chicken-breast, which scarcely passes for a deformity, is often sufficient greatly to alter the relation of the heart to the walls of the chest. It often thrusts it forward, and brings its whole anterior surface in contact with the sternum and ribs. Hence in such cases the question, whether the heart be sound or unsound, becomes puzzling enough. Sound or unsound, its impulse is to be felt and seen in all the space at which it lies in contact with the chest, and the same space is dull to percussion. Extensive præcordial impulse and extensive præcordial dulness are the very signs of hypertrophy; and if to these be superadded the endocardial murmur, you have the complete signs denoting the commonest form of complex unsoundness which the heart is apt to undergo, viz. hypertrophy with valvular disease. But beware, now especially, beware, of creating the endocardial murmur by the application of the ear or the stethoscope to the præcordial region. Nothing is easier. I have done so frequently in such cases by way of experiment." . 64.

Dr. Latham mentions a circumstance touching the history of Phthisis, that—if the accuracy of the remark be confirmed by the experience of other medical men—is of the highest importance, and therefore requires to be generally known. To prevent all mistake, we shall give the description in his own words:

"Fancy a line drawn from the left side of the sternum along the upper edge of the second costal cartilage, and continued an inch along the second rib; and another line drawn from the sternum along the lower edge of the third costal cartilage, and continued an inch along the third rib. Between these two lines a space is included, in the whole or in part of which a murmur is often audible coincident with the systole of the heart, when no such murmur can be perceived either in the præcordial region, or in the course of the aorta, or in the carotids, or in any part of the arterial system, but here and here only. It is a gentle bellows-murmur, quite obvious to the ear, and unmistakable in its character.

"Of such a murmur, often audible in this situation exclusively, I am certain as a matter of fact, and certain too of its very remarkable accompaniments. I have witnessed it either in those who were undeniably consumptive, or in those who were too justly suspected of being so. I cannot say in what proportion of the phthisical it occurs; but I am continually meeting with it." 66.

He cannot offer any satisfactory, or even plausible, explanation of the phenomenon. Some one may conjecture that it may be seated in the pulmonary artery.

We must not dismiss this part of our subject without reminding the reader that incipient Valvular Disease of the Heart may exist without there being any permanent endocardial murmur; for this may be perceptible, only when the circulation is somewhat quickened. Neither the cardiac impulse nor the sounds may be at all altered, as long as the person is quiescent; but after motion, the one will generally be found to be stronger, and the other to be more or less distinctly modified, if any serious lesion of the valves be present. Hence, whenever we have reason to suspect from the general symptoms the existence of cardiac disease, the utility of making the patient walk once or twice briskly round the room—or, what is still better, go up a flight of stairs rather quickly—and of then examining the state of the heart's actions and sounds, should there be any grounds for ambiguity in the diagnosis. We proceed now to particulars.

*Endocarditis.*—It is well known that it was Dr. Latham who first pointed out (in 1826) that, whenever the heart is affected in acute Rheumatism, a sound different from the sound of health is always heard to accompany its contractions. His idea then, and for some time afterwards, was, that this abnormal sound was somehow or other connected with an inflammation of the *pericardium:* in course of time, however, he began to suspect that it proceeded from the internal lining of the heart. "But for years the practice of this great hospital did not afford me a single opportunity of resolving my doubt, or of confirming my conjecture. For of that disease of the heart, which, coming on during acute rheumatism, is characterised by the bellows-murmur, no patient of mine ever died, and I could learn nothing about it from dissection. But what my own experience would not furnish, M. Bouillaud's has supplied. Many have died during the active progress of this disease under his care, and dissection has found it to be inflammation of the endocardium. Thus we are indebted to M. Bouillaud for our first knowledge of this important fact."*

Nearly about the same time (1836), the attention of the profession was directed, by Drs. Watson and Stokes more especially, to the friction (or *exocardial*) murmurs, as characteristic of pericardial inflammation. These sounds always suggest the idea of attrition, as if produced by two surfaces moving to and fro upon each other; whereas, on the other hand, the *endocardial* as invariably suggest the idea of a blowing or rushing, or whizzing noise. The former are caused by the surfaces of the pericardium becoming either unusually dry, or rough and irregular by the deposit of lymph; the latter by the narrowing of the orifices through which the blood has to pass from one cavity to another. When both descriptions of murmur are found to be present in a case of acute rheumatism, we have the strongest presumption for believing that there is an inflammatory state both of the Pericardium and the Endocardium. We are not inclined to go quite so far as Dr. Latham does, and assert that "the bellows-murmur coming on in the course of that disease is a sure sign of endocarditis;" because we know that this murmur is apt to occur, especially in persons of an irritable temperament, after copious depletions of blood or any great lowering of the system. Still, it is a most valuable symptom, and it therefore becomes a matter of first-rate importance, that its early development should be ascertained. The following emphatic remarks will arrest the attention of every one who reads them.

"In endocarditis, besides the endocardial murmur, there may be other symptoms present directly referrible to the heart, or there may not. There may or

---

* The reader will find a succinct account of M. Bouillaud's opinions in our review of his work.

may not be pain. There may or may not be an excessive impulse, or an intermittent, irregular, or fluttering action of the heart. But the fact of endocarditis is not rendered more or less certain by their presence or absence.

"There may be both pain and palpitation; yet endocarditis cannot be surely inferred to exist, unless there be the endocardial murmur withal. There may be neither pain nor palpitation, yet endocarditis cannot be inferred not to exist, if the endocardial murmur alone be present.

"Seeing, then, that the endocardial murmur alone can determine the existence of endocarditis, you are required to search after it in every case of acute rheumatism. I say emphatically *to search after it*, because it is one of those signs which must always be sought before it can be found. It does not intrude itself upon our notice like palpitation, or an irregular pulse. The patient does not draw our attention to it as he does to pain. The physician must make it out entirely for himself. And indeed it is infinitely important that he should have the earliest possible notice of it with a view to the earliest possible application of the remedy." 105.

The occurrence of the Endocardial murmur, in cases of rheumatism, is often preceded, for one or two days, by a very perceptible change or modification of the first or systolic sound of the heart: this is generally much longer and rougher than in a state of health. In the majority of instances, however, the change from the natural sound to the endocardial murmur takes place without any such notice or prelude. The exact seat of the murmur, and the direction in which it seems to be prolonged, must be taken into account to enable us to determine which of the orifices of the heart is (most probably) affected.

With respect to the rational symptoms of the earlier stages of Endocarditis—pain in the præcordia, increased perhaps upon a deep inspiration and by pressure, dyspnœa, a sense of anguish about the region of the heart, an increase in the force of its impulse, and an irregular, perhaps intermittent, pulse—it should be borne in mind, that in some cases they are sufficiently well marked before any trace of endocardial murmur can be heard; while in other cases the auscultatory precede the development, or at least the distinct announcement, of the rational symptoms. The following remarks, on the value of *pain* as a diagnostic sign, well merit the attention of the practical man.

But of all symptoms mere pain is the most inconstant and uncertain, whatever be the disease. It is so in pericarditis. It is present in one case, and absent in another strangely and unaccountably. I have known much pain, when the disease has been of little severity, of short duration, and of easy cure: and I have known the severest pericarditis pass through all its stages without pain. All other symptoms have been present to mark its reality and its progress: the murmur and the præcordial dulness, and the fluttering heart, and the respiratory anguish. And sometimes the patient has died, and sometimes he has escaped by a tardy and precarious convalescence. But from first to last there has absolutely been no pain.

"Do not be surprised at this. Pleurisy may exist without pain; even acute, rapid, pus-effusing pleurisy. Peritonitis may exist without pain; even acute, rapid, pus-effusing peritonitis. And so, too, if in pericarditis there is sometimes no pain, it fortunately happens that there are other signs by which we can fix our diagnosis of the disease equally well without it." 142.

*Pericarditis.*—As the *bellows-murmur,* when it occurs in a case of acute

Rheumatism, is the pathognomonic auscultatory symptom of Endocarditis, so is the rubbing or *attrition murmur* (heard in the cardiac region) the pathognomonic sign of Pericarditis. The peculiar character of the latter sound differs very much in different cases; hence the variety of similitudes to which it has been compared. It not unfrequently resembles the noise produced by rubbing the hands together, or the cuffs of one's coat upon each other, or two pieces of rough paper; at other times it is like that caused by the crumpling of parchment, or the creaking of shoe-leather, or the churning of milk, &c. &c. As Dr. Latham suggests, it would be much better to discard, or at least not to use generally, any word derived from these supposed resemblances, and to adopt instead the generic application of *exocardial*—intimating thereby that the sound in question proceeds from the external surface of the heart.

Along with this abnormal auscultatory sign, there is very generally associated an increased dulness on percussion, and this too over a greater space than usual. Occasionally—but this is a rare occurrence—the exocardial sound, after having been for two or three days distinctly perceptible, has ceased altogether and perhaps has again returned. Such a circumstance may be owing to water, which had been effused into the bag of the pericardium, becoming absorbed under the influence of the remedies employed. Besides the more important symptoms of an exocardial sound and increased dulness on percussion, there are two other physical signs in pericarditis, mentioned by our author as being "directly referable to the heart, indicating the same conditions of disease, and often found in their company." These are described in the following words:

"In pericarditis, while the præcordial region is dull to percussion and the exocardial murmur is heard, an undulating motion often becomes visible to the eye in some of the spaces between the cartilages of the ribs on the left side. It has always been either between the cartilages of the second and third ribs, or of the third and fourth, or between both at the same time, that I have seen this motion, and never in any other situation.

"So, too, in pericarditis, while the præcordial region is dull to percussion and the murmur is heard, a vibratory motion often becomes sensible to the touch in some of the spaces between the cartilages of the ribs on the left side. As I never *saw* the undulatory, so I never *felt* the vibratory, motion elsewhere than either between the cartilages of the second and third, or of the third and fourth ribs, or between the cartilages of both simultaneously.

"The vibration (I believe) is the more frequent of the two, and often occurs unaccompanied by any visible undulation. But the undulation was never apparent to my eye without my finger being able to detect a sensible vibration at the very same spot." 134.

It is to be remembered that these latter signs are by no means of constant occurrence in Pericarditis; in many cases, neither of them is ever discoverable; and, when they are present, it is never in the early stage of the disease. Moreover, the vibratory movement in certain of the intercostal spaces is occasionally met with in other cardiac maladies; as, for example, in disease of the semilunar valves of the aorta and pulmonary artery.

We now proceed to lay before our readers some valuable statistical data, derived from Dr. Latham's practice at St. Bartholomew's Hospital, to shew the intimate connexion between Endocardial and Pericardial inflammation on the one hand, and the existence of acute Rheumatism on the other.

In the course of five years, from the beginning of 1836 to the end of 1840, there were admitted under our author's care, 136 cases of acute rheumatism. Of these, 75 occurred in males, and 61 in females. Of the 75 males, the heart was affected in 47; and of the 61 females in 43. Now in these 47 cases, the seat of the cardiac disease was as follows: in 30, the endocardium alone was affected; in 3, the pericardium alone; in 7, both membranes were inflamed; and in the remaining 7 the exact seat of the disease was uncertain.

Of the whole number of males, in whom the heart was thus variously affected, only 3 died; and in these 3 cases, both the Endocardium and the Pericardium were affected.

Of the 43 cases of rheumatic heart-complication in females, "the seat of disease was the endocardium alone in 33; the pericardium alone in 4; and both endocardium and pericardium in 4; and the exact seat of the cardiac disease was doubtful in 2.

Of the whole number of females in whom the heart was thus variously affected none died."

Here then we see that, in *two-thirds* of these cases of acute Rheumatism, there was some affection or another (certainly of an inflammatory nature) of the heart. Before the publication of Bouillaud's Researches,* it was universally supposed that it was the pericardium that was generally affected. The fallacy of this opinion is abundantly shewn by the data which we have just now given. From them "it appears that Endocarditis occurs nine times in acute rheumatism, for pericarditis once; that simple endocarditis constitutes more than two-thirds of all rheumatic cardiac affections, and simple pericarditis only one-thirteenth; and that pericarditis is more frequently found in combination with endocarditis than alone."

It will be observed that not one of the 63 cases, in which the endocardium alone was affected, proved fatal. From this it might be inferred by some that Endocarditis is any thing but a very formidable malady. But then let it be never forgotten by the practising physician that the disease often leaves behind it, *when seemingly cured*, the rudiments of future mischief; and that the complete cessation of the endocardial murmur occurred in but a small proportion of the cases (17 out of 63) when they left the hospital. As long as this auscultatory phenomenon lasts, we cannot fairly regard the heart as completely restored to a state of health. In the seven cases of simple Pericarditis, life was saved in all; and moreover no trace of exocardial murmur remained, after the convalescence was fairly established. From the circumstance, however, of the continuance, in four of of them, either of a certain degree of cardiac uneasiness, or of an increased impulse of the heart, or of some alteration in its natural sounds, it was

* The reader will find tolerably ample reviews of this gentleman's work on Diseases of the Heart, and of his *Nouvelles recherches sur le Rheumatisme articulaire aigu en general, et specialement sur la loi de coincidence de la Pericardite et de l'Endocardite avec cette maladie*, in the Numbers of this Journal for April and July, 1836.

considered highly probable that adhesion, to a greater or less extent, had taken place between the opposite surfaces of the inflamed membrane. If such were the case, there is too much reason to fear that the pericardial adhesion would be the ground-work and "point de depart" of a more serious cardiac lesion at some ulterior period of life.

In the three fatal cases, the auscultatory signs denoting inflammation of the endocardium and pericardium were well-marked, and on both membranes dissection disclosed the recent effects of inflammation. We give the details of the *post-mortem* appearances in one: "the folds of the pericardium were universally adherent, but were easily separated. The connecting lymph was peculiarly vascular over the left ventricle, and being detached, discovered some spots upon the surface of the heart which looked like pus. The endocardium bore marks of inflammation on both sides of the heart. On the tricuspid valve, at a little distance from its free edge, was a spot, one-third of an inch in diameter, pink in the centre, and surrounded with a white elevated border. On the mitral valve were small pearly bodies, about the size of millet seeds, fringing its free edges. The aortic valve was thickened and of a pinkish colour, and displayed upon its surface, a little below its free edges, bodies of the same form and size as those found upon the mitral valve. The general bulk of the heart and the capacity of its cavities were natural, and its muscular substance had the appearance of health."

In one of the other cases, there was not above two or three drachms of fluid in the pericardial sac, which, although invested with curd-like lymph, was not at all adherent; whereas, in the third, there was nearly three ounces of fluid, the pericardium being only partially adherent to the surface of the heart by loose bands of lymph.

It is not easy to determine whether it is the Pericardium or the Endocardium that becomes first inflamed in these compound cases. In one instance, the exocardial and the endocardial murmurs arose *simultaneously* after the patient's admission into the hospital: and in 2, they were found already co-existing at the time of admission. In 4, the endocardial was prior to the exocardial murmur; and, in 4, the exocardial was prior to the endocardial murmur.

Dr. Latham's ninth lecture is devoted to the consideration of a point in the history of acute Rheumatism that has not hitherto met with the attention it deserves. While every physician in the present day knows full well that pericarditis and endocarditis are very common accompaniments of this disease, not many, probably, are aware that inflammation of the lungs—we use this term to comprehend pneumonia, pleuritis, and bronchitis—is also a not unfrequent complication at the same time. Of Dr. L's 130 cases of acute rheumatism, the heart was inflamed in 90, or in two-thirds of the whole; while the lungs were inflamed in 24, or one in $5\frac{1}{2}$. These 24 cases consisted of 4 of Bronchitis, 18 of Pueumonia, and 2 of Pleurisy. In all, the pulmonary disease was of a serious character. "In the four instances of bronchitis, the affection was no mere catarrh, but an inflammation largely diffused through both lungs, producing deep oppression and dyspnœa. Of the two pleurisies, one was single and the other double. The single pleurisy produced a large effusion into one side. The double pleurisy produced a double hydrothorax. Of the 18 instances of pneumonia, in 9 the disease was of one lung, and in 9 it was of both."

Whence this highly dangerous character of the pulmonary disease? From the circumstance, most probably, of its being generally associated with cardiac inflammation. For of the 24 cases, 5 only occurred in the 46 cases of rheumatism in which the heart was unaffected; whereas, of the 90 cases in which the heart was inflamed, the lungs were also inflamed in 19. Again, of these 19 cases, no fewer than 8 of them occurred in patients in whom Endocarditis and Pericarditis existed simultaneously; and, as the number of such patients did not exceed 11 in all, we see how frequently a pulmonary complication is apt to occur under these circumstances. Of the remaining 11 cases, 7 occurred in the 63 cases in which the endocardium alone was affected; and 4 in the 7 cases in which the pericardium alone was inflamed.

From these data, we may fairly infer that it is chiefly in those cases of acute Rheumatism in which the pericardium, either alone or simultaneously with the endocardium, is affected, that we have to dread the supervention of pulmonary inflammation.

Dr. Latham has related at considerable length two cases of this complication of inflammatory affections, which we could have wished to have analysed and commented upon; but our limits prevent our doing this, and we therefore pass to another interesting point in the history of cardiac inflammation, to which we have not as yet alluded.

Although by far the greatest number of cases of Endocarditis and Pericarditis occur in connection with Rheumatism, there is no doubt that these diseases are not unfrequently met with, associated with other morbid states of the system. Hitherto, it must be confessed, not much progress has been made in this field of pathological enquiry. A tendency to cardiac inflammation seems to exist in various orders of the Pyrexiæ; more especially in some of the eruptive fevers, Scarlatina and Measles to wit. Certain it is, that in neglected or injudiciously treated cases of these exanthemata—Hooping-cough may be added to the number—we very frequently meet with symptoms of decided Cardiac disturbance. We are surprised to find that Dr. Latham has not alluded to this circumstance; nor has he noticed another, rather important, fact connected with the ætiology of heart-disease; viz. that a gouty disposition seems not unfrequently to have something to do with the development of valvular and other lesions of this organ. He has however pointed out some conditions of the system, unconnected with rheumatism, in which Endocarditis and Pericarditis have been found; and to these we now invite the reader's attention.

In certain cachectic states, when the powers of life have become exceedingly reduced from want, exposure to the weather, neglected ailments, and so forth, it would seem that inflammation of the heart is sometimes set up; the symptoms, however, indicating its development being all the while very obscure. The following observations by our author suggest some important reflections:—

"It would hardly be suspected that the very act and process of dissolution could give occasion to *new* disease. But such is the fact. And it happens especially, if the dissolution be slow and lingering; and then this new disease is often even of an acute kind. In no part of the body is this new disease more apt thus to light up, at the very going out of life, than in serous membranes. Among phthisical patients, who have been dying by little and little for many weeks, the instances have been numerous, in which upon dissection I have found the marks of very recent peritonitis, the cavity of the abdomen containing a whey-like fluid, and the surface of the intestines covered with flocculent lymph, and streaked with red, and adherent where their folds lie in contact. Yet in many such cases the peritonitis has given no notice of its existence during life by its proper symptoms, and after death has occasioned great surprise by its discovery. And thus, too, pericarditis will arise when the system is at its lowest state of depression. I have known some instances (and others have been reported to me) where, after severe accidents and severe surgical operations, the powers of life being brought very low, and existence with difficulty maintained during some days, upon death eventually taking place, the pericardium has been found covered with flocculent lymph, and its cavity distended with serum mixed with pus and blood. These were the products of the most acute inflammation. But the patients were scrupulously watched during life, yet no symptoms indicative of inflammation were discovered." 357.

Several cases are related in illustration of these observations.

Dr. Latham remarks, that in all the cases of Pericarditis unconnected with rheumatism, which have come under his notice, it was invariably complicated with a morbid state of other internal parts, and especially with disease, or the results of disease, of similar structures; as with inflammation, or with serous and sanguineous effusion, of the pleura or peritoneum: in a few cases, the brain or its membranes are affected. He quotes from the work of Corvisart several cases, which quite accord with this statement. Of six cases of pericarditis not in alliance with rheumatism, recorded by Andral, three were complicated—either with tubercles and vomicæ, or with asthma and bronchial congestion, or with petechial small-pox—and three were uncomplicated. Of these latter, one commenced with symptoms of fever and cerebral congestion: subsequently severe pain in the cardiac region, accompanied with tumultuous action of the heart, came on; the patient recovered. The next one was less fortunate; the symptoms were violent and proved fatal on the sixth day. On dissection, the pericardium was found distended with a sanguineous fluid, and its surface coated with reddish membranous concretions.

Having thus, at very considerable length, examined the history of Endocardial and Pericardial inflammation, more especially in reference to its symptoms and its predisposing causes, the question naturally arises, what is the more immediate cause of the disease, and what peculiarity is there in Rheumatism for example, or in Scarlatina, that should be liable to induce an inflammatory affection of the Heart, rather than of any other organ? For our own part, we have long been inclined to believe that it is to a state of the circulating fluids that we must look for an explanation of this pathological phenomenon. Is it not reasonable, to say the least of it, that an altered condition of the blood may occasion an irritation of the lining membrane (more especially) of the heart? That such an alteration exists in the diseases which we have named—Rheumatism and Scarlet Fever—will be denied by none; and may not the same be said of the other states of the system, with which active cardiac disease has been observed to co-exist? The *ætiology* of Heart-affections is yet but very imperfectly made out. In the general run of cases, it will be difficult or utterly impracticable to ascertain the probable cause. In a good many, indeed, it may be found that the patient has, at some period or another, suffered from Rheumatism; but certainly not in the majority. Let us hope that the great attention that is now paid to humoral pathology will, ere long, throw some light upon this subject.

These few remarks will form an appropriate preamble to our introducing Dr. Furnivall and his work to the acquaintance of the reader. The following passage in the Preface may be regarded as enunciating the theme or position which Dr. F. has proposed to himself for elucidation, and as explaining at the same time the motives which have induced him to hazard the perils of authorship. It is necessary to premise that the language of our author is, on many occasions, far from being very distinct or even intelligible, in consequence of the repeated violation of the ordinary rules of syntax and punctuation. After telling us that he has long avoided speculative enquiries, and devoted his attention to practical subjects, he proceeds:—

"But, after lengthened experience, accumulated facts, and a consideration of the juvantia, forced on his mind a rationale of causation—a theory—which, if hereafter established as correct, must, he thinks, lead both to a clear understanding of the at present obscure pathology of Rheumatism, and to an effective protection against Heart-disease, as its consequence; it must, therefore, conduce to a great curtailment of mortality, and to a prevention of much misery, more especially in the Poor Man's case." v.

The exposition of this *theory*, and of its important practical consequences, is thus laid down: the extract is a long one; but it is best to give the pith of a book in the author's own words when, we can.

"Most authors attempt to explain the liability to heart-disease during a rheumatic attack, by referring to identity of structure. Rheumatism, they say, affects the fibrous structure in preference, and fibrous structure abounds in and about the heart. Now this attempt at explanation might be received as a solution of the difficulty, if rheumatism were the only cause of heart-disease; but this is not the case; nor can we thus explain the well-known tendency to inflammation of the heart's membranes, which exists in scarlatina, measles, &c. These diseases do not attack the fibrous structure exclusively, nor at all. In two cases I have found pertussis to have been followed by or accompanied with pericarditis and cardiac hypertrophy; and scarlatina, as a cause, is by no means unfrequent.

"The only proximate cause adequate to the effects, seems to me to be a morbid state of the blood itself; accordingly an excess of fibrine has been detected in the blood of rheumatic persons; which excess must render it highly and morbidly stimulant. Probably there is also some other arrangement of its primary components; which, in the present state of our knowledge, may be best described by saying, that it is relieved by an elimination of acid through the secretions, and best obviated in treatment by the use of alkalies. Possibly lithic acid may exist in excess in the form of lithate of soda. Whether this hypothesis be correct or not, I hope to be able, at some future period, to shew evidence of the paramount utility of alkalies in the prevention of cardiac disease in rheumatism; and I can aver, that wherever I have seen alkalies given in rheumatism, judiciously combined with other remedies, there has been no distressing heart-complication; and I have read of cases where life has been lost, through cardiac disease from rheumatism, in spite of a treatment exceedingly able and energetic, except in the single omission of alkalies. It has been attempted to refute the hypothesis, by the answer that the blood will not allow free acid in its composition; that it is an electro-negative body. But it is not necessary that the acid admixture should be a free acid; on the contrary, it may exist under some other form; and probably in the serum. Lately I have been pleased to find that this conjecture, as to the probability of an acid nature of the blood in rheumatism, is not confined to myself; for I find an eminent practical physician, Dr. Schönlein, of Vienna, to be of this opinion also. He mentions a case where an ulcerated surface overspread itself during the night, with an earthy crystalline crust. According to Dr. Simon's researches this concretion of pus contained uric acid in a remarkable degree, combined with soda and ammonia. Dr. Schönlein thus expresses himself. 'This is quite a new fact, which, formerly, indeed was conjectured, but which has now been confirmed by chemical researches. To judge from this circumstance, it is not a very remote inference to suppose that uric acid is also existing in the blood in this disease, it being also ascertained that it also occurs in great quantity in the urine, as also in the perspiration. It has been now, moreover, found in the secretion of rheumatic ulcerations; it most likely occurs in the secretion of the mucous membranes (for the saliva has also an acid reaction); it probably will therefore soon be discovered in the blood of such patients.—*The Medical Times.*'

"So think I." 20.

Dr. Furnivall subsequently informs us that, since he has been in the habit of treating Rheumatism by the alkaline method, he has not met with a single instance out of at least 400 cases, where any Heart-disease was consecutive upon it. The alkalies act, he says, in a two-fold manner; viz. by "diminishing the undue quantity of the Fibrine proved to exist in rheumatic blood," and also by "a direct chemical action on the abnormally acrid state of the blood and the secretions."

There is another claes of remedies, or at least one member of this class, that Dr. Furnivall most urgently and perseveringly recommends to the attention of medical men, in the treatment of cardiac disease—we allude to the class of sedatives; and of these to *aconite* in particular. This potent drug is said not only to have the power "of effecting a somewhat lasting modification of the heart's irritability," but also to act as a direct antiphlogistic or subduer of inflammatory action, and as a corrigent of a highly fibrinous state of the blood.

"Now any remedy which will prove an effective substitute for the detraction of blood, will be found nearly invaluable in certain cases of heart-disease—namely, in all such as occur in weakly persons, and yet are accompanied with great excitement of the circulation. In such delicate habits of body, there are two great sources of danger—the one arising from the very rapid progress of the disorder, unto, perhaps, irremediable changes of the delicate structure affected, and the consequent necessity for adopting the most energetic measures of depletion, &c., to counteract; the other, arising from the patient not having strength enough to bear the exhaustion resulting from the active treatment which has been adopted; so that although the medical man may have the satisfaction of witnessing the reduction of the disorder; he yet may find his patient shortly after attacked by tubercular consumption, or some other disorder. Under such circumstances, Aconite will be found the very substitute we want; and a proper trial will soon convince that we may thus avoid detracting many an ounce of blood which otherwise must flow." 30.

Our author does not hesitate to assert that, in future, he expects to meet with few instances even of severe acute Rheumatism, in which the judicious exhibition of the aconite may not entirely supersede the use of venæsection ! It is a direct antiphlogistic, not only by its sedative action upon the heart and arteries, but also by its power of defibrinating the blood. The only case, however, which he adduces to prove the powers of his favorite remedy upon the buffy state of the blood, must be regarded as utterly inconclusive in the eyes of any dispassionate reader : it surely cannot satisfy Dr. F. himself. The case was one of Aortic Aneurism : the patient had been repeatedly bled, and the blood drawn had always exhibited a buffy coat. He came under the care of Dr. F., who gave him aconite and *blue-pill;* he was subsequently bled; no intimation however is given of the lapse of time since the preceding venæsection, although it seems to have been a month or two ; the blood this time was not buffy; *ergo*, the aconite produced the change.

After this statement of Dr. Furnivall's general therapeutic views, we shall be better prepared to appreciate the value of the modes of treatment, recommended by him and Dr. Latham, in inflammation of the endocardium and pericardium. It is quite unnecessary to allude to the description of these diseases given by the former gentleman; for we regret to say that it is most lamentably defective, and utterly unworthy of any one who has studied their history—we shall not say by the bed-side of patients, but even from the writings of recent authors. Statements of the most incongruous and conflicting nature, every now and then, occur withint he compass of a single page, so that the reader is repeatedly inclined to ask whether the author has ever really seen the disease which he is describing. It is quite obvious that Dr. F. is exceedingly little acquainted with the discovery and application of auscultatory knowledge in the diagnosis of cardiac diseases, more especially of Endocardial and Pericardial inflammation. We had marked several passages for censure ; but it may be unnecessary to specify them, as the third and fourth chapters ought to be entirely re-written, if the work be so fortunate as to reach a second edition.

There is, however, one offence against fair play which we cannot pass over without a special rebuke. Dr. Furnivall is, on too many occasions, exceedingly negligent in not giving any exact reference to the authors whom he has honoured by quoting from their works. Several quotations are made without any notice whatsoever of the source whence they are derived. For example, we find a page and a half taken, word for word, from the number of this Journal for July 1844, and the passage is not even placed within inverted commas. This is not as it ought to be. The extract indeed is a translation from a French work; but it would have required, we should think, a mesmeric *rapport* to have existed between Dr. F. and ourselves, before both could have made use of exactly the same words in translating one language into another.

So much in the way of censure ; now for a more pleasing duty.

The therapeutic directions of Dr. Furnivall are, on the whole, much more commendable than his descriptions of the phenomena of disease. As we have seen, he keeps steadily in view the abnormal condition of the blood and of the urinary secretion in the *treatment* of acute Rheumatism, and of the dangerous complications which so frequently attend it. In this respect he has greatly the advantage of Dr. Latham, who seems to have nearly quite overlooked these two most important indications of practice in all cases of rheumatic disease. We may rest assured that, as long as the blood remains charged with an excess of its fibrinous constituent, and the urine is scanty and deep-coloured, there will always be a strong tendency to the development of some internal mischief, and to the persistance of what already exists. Our practice should therefore never fail to be directed to the correction of these two morbid states, and we shall find that those very remedies, which are known to have the most decided effect in defibrinating the blood and in neutralising the acidity—while at the same time they promote the quantity—of the urine, are unquestionably among the most powerful anti-rheumatics which we possess. That blood-letting and mercury are the best for the first of these objects, few will be disposed to question; and none can deny the superior efficacy of alkalis for the attainment of the second.

Dr. Latham has devoted no fewer than six entire lectures—upwards of a third of his work—to the treatment of acute Rheumatism and its cardiac complications. His practice, we may remark, is rather bold and heroic, than comprehensive and discriminating. He first canvasses the comparative merits of venæsection, opium, and purgatives (large doses of calomel at bed-time followed with black-draughts in the morning), employed singly and apart from each other, and then points out the advantages and disadvantages of each of these remedies. Several of his remarks in this estimate are far, we think, from being judicious. Thus, in his introductory observations, he says that the practice, pursued by some physicians, of taking a single symptom or set of symptoms—for example, the high vascular action, or the pain and nervous disquietude, or the disordered state of the intestinal secretions—and of making all their remedies to bear upon that one point, is in many respects an exceedingly good and useful plan.

"Let me repeat my testimony to the success of this practice in acute rheumatism; the practice, namely, of choosing some single indication, and steadily pursuing it to its fulfilment. It is a very rational practice. It is founded upon experience, and it compasses its end by very simple means; and the manner of its successful operation may be well conceived, if it cannot be entirely explained, in the present state of our knowledge. Disease is a series of new and extraordinary actions. Each link in the series is essential to the integrity of the whole. Let one link be fairly broken, and this integrity is spoiled, and there is an end of the disease; and then the constitution is left to resume its old and accustomed actions, which are the actions of health." 187.

There is a good deal of fallacy, if not of positive and very serious error, in such reasoning as this. Disease is not a simple chain of single *links;* but a twisted rope of many *strands.* You may break or cut one; but the rope still holds together, until the force applied makes all give way. And so it is with an active disease like acute Rheumatism. There is more than one series of morbid actions to be corrected; and therefore there is more than one curative indication to fulfil. On this ground alone, we have always been unwilling in our own practice to adopt that mode of treatment which has been recommended by many excellent practitioners, and which Dr. Latham tells us he generally followed in the earlier years of his hospital practice—we mean that by the free use of opium; for example, a grain every eight, six, or even four hours, until a decided impression is made upon the malady. The pain of the disease may indeed be abated, and the system may thus be tranquillised and kept quiet, while it is engaged in eliminating the *morbid materials* that unquestionably exist in the blood and the secretions derived from it, either by the skin or by the kidneys, or by both; but surely the opium can not have any *direct* effect in diminishing the excess of fibrine in the blood, or in neutralising the superabundance of uric acid and urea in the urine. Rheumatism, to be treated scientifically, must be regarded as (in part, at least) a *humoral* disease, and as affecting the fluids even still more than the solids of the body.

After weighing the relative value of the three modes of practice—free Venæsection, Opium, and Purgatives—Dr. Latham, like every sound physician, comes to the conclusion that it is wiser and better to have recourse to all three (in moderate doses) simultaneously, than to trust to any one of them apart and singly, more vigorously pursued. What has surprised us a good deal is that he does not allude to the exhibition of mercury—not as a cholagogue or purgative, but as an alterant or *mercurial*—in the treatment of uncomplicated acute Rheumatism; and yet surely, as this is the great remedy in endocardial and pericardial inflammation,—which he has shewn to be present in the majority of cases—we might have expected that he would have recommended it for general adoption. For many years past, we have invariably exhibited moderate doses of mercurials (with or without opium, according to circumstances) in the treatment of acute rheumatism, not omitting the simultaneous use of occasional blood-letting, and of purgatives and alkaline salts; and we have every reason to be satisfied with the practice.

As a matter of course, whenever there is reason to suspect the co-existence of cardiac inflammation, our treatment must be, if not more active, at least more watchful and sedulous. Local blood-letting, by leeches on the front of the chest and cupping between the spine and edge of the left scapula, is of great importance, if the system has been sufficiently lowered by general bleeding. Dr. Furnivall is of opinion—and he is quite right—that the use of depletory means may be carried much farther with safety in Pericarditis than in Endocarditis.*

"In the latter, syncope might be fatal; and we therefore must use the lancet very cautiously; while in Pericarditis we may bleed with much boldness. Again, nausea unto faintness will prove beneficial in the latter; but in the former, where the fibrine renders the current of blood through the heart very sluggish, faintness from languor may favour deposition of fibrinous concretions; and the patient may thus be precipitated into the second stage of fatal obstruction, which has been described." 63.

---

* Dr. Latham also appears to be of the same opinion; for, after noticing the much greater fatality of endocardial inflammation in M. Bouillaud's practice than in his own, he goes on to observe:

"Now M. Bouillaud's treatment of endocarditis has always been vigorously antiphlogistic. He has employed large and repeated bleedings, and all other remedies calculated to control inflammation, except mercury. Mercury he never used.

"My treatment of endocarditis, on the other hand, has not been vigorously antiphlogistic. I have seldom employed venæsection at all, and never largely. But mercury has been among my remedies in almost every case."

For the same reason, he would never administer Digitalis in any case of endocardial inflammation. "Alkalies," he goes on, rather incautiously, to say, "may be considered as almost specifics in this disease; and seem to reduce the abnormal thickness of the blood, very speedily and very effectually. By the combined action of mercurials, aconite, and alkalies, it appears to me that the slaughtering depletions which have been recommended, may be dispensed with; and dilatation of the heart, general exhaustion, tubercular consumption, &c., may be avoided."

We need scarcely say that blisters and other counter-irritants,* applied over the region of the heart and between the shoulders, are of very great value, when the more active and pyrexial symptoms of cardial inflammation have been subdued. Dr. Latham has strangely omitted to make mention of them; nor does he allude to the use of the Hydriodate of Potash, which has been so generally recommended as an adjuvant of mercury in so many sub-inflammatory diseases. Should any sudden attack of severe pain and dyspnœal anguish come on, in the course of any cardiac disease, a full dose of opium is the proper remedy.

So much of our space has been occupied with the consideration of the two "primitial or primigenial" (to use an expression of Dr. Furnivall) diseases of the heart, that we shall only very briefly notice the other numbers of the series.

Of *Hypertrophy*, we are informed by him that there are two kinds—"the one, a simple affection without any morbid productions, and with but little augmentation of tissue; the other is caused by some mechanical obstacle in the course of the circulation."

He repeatedly speaks of *functional* Hypertrophy, and occasionally too of *functional* Dilatation—expressions which it is certainly not very easy to understand. There may be *functional* diseases, whose symptoms simulate those of diseases which are of an *organic* nature, and which depend upon an actual alteration of structure; but might we not almost as well talk of a functional Exostosis, as of a functional Hypertrophy? In his account of the *treatment* of this very common cardiac disease, Dr. F. has altogether omitted to mention by far the most important (in our opinion) remedy—we mean, a *seton* over the region of the heart. According to our experience, it is worth all the internal remedies that have ever been recommended. Without it, not much good can be done; and from it, we may generally look for very *decided* benefit; provided, at the same time, the various secretions be attended to, and over-exercise of the body be avoided.

With regard to the *treatment* of Dilatation, is Dr. Furnivall justified in attributing the practice, which he very properly condemns, to any medical man in the present day? "The treatment of Dilatation is very obvious; we have to try to restore strength and vigour to the affected ventricle, to obviate palpitations, and relieve secondary or consequent symptoms. The routine of V. S., Digitalis (which is almost certain to be prescribed), and of debilitating drugs, is highly objectionable; nor should a low un-

---

* Dr. Furnivall rather strangely says that "hot spirits of turpentine, laid on by old linen (!), will be preferable to blisters, if we want to use the stethoscope frequently."

nutritious diet be allowed in any case of Dilatation whatever; yet such a routine used at times to be met with, perhaps on account of the quick pulse and the dyspnœa."

He suggests, rather than distinctly recommends, a trial of Strychnine in certain cases of dilatation: we much question the propriety of such an experiment.

Several of Dr. F.'s illustrative cases of this form of cardiac disease, especially those which occurred in young females, may fairly excite, in the reader's mind, some doubts as to the accuracy of the diagnosis that was formed. Let us take the following example:

"Jan. 14, 1841.—Clara P., ætat. 24, been ill twelve months with palpitation and cardiac disturbance; cannot exert herself; amenorrhœa for the last three periods; and there are no signs of any nisus menstrualis; respiratory murmur good; but the heart acts with too much noise and impulse over a large space; a whiffing sound, not a bellows-murmur, with the first sound, to the left of sternum; a pain near left nipple; pulse 108, and rather full; been getting worse gradually, but latterly very sensibly so; skin not cold; can lie on right, rather that on left side.

"Hirudin. x., part. dolent. Empl. Canthar. sterno et thorac. dextro. ℞. Submur. Hydrarg. gr. xviij., Extr. Aconit. gr. iij., Pil. Scill. C. ℈j. m. ft. pil. 12. Cap. j., nocte maneque. A mixture of Bicarbonate of Soda with Hydrocyanic Acid. Jan. 19. The Mercury was omitted; the cardiac phenomena much relieved. Jan. 23. Caught cold; and there was a return of impulse, with a cough and pyrexia. Eight leeches to sternum, and add ¼ gr. Extr. Aconit. to each dose of the mixture. At night, a Pil. Sapon. C., with Ipecacuan. and Extr. Col. C. By the 28th the undue impulse was diminished, and there were no palpitations. On the 11th of February she was well enough to be discharged, but cautioned as to her conduct. She took with her Aloetic pills to induce the catamenia, which afterwards came on. This person was becoming worse; and the catamenia having stopped, the blood becoming more and more deteriorated, would have increased the cardiac Dilatation to a fatal extent ultimately." 115.

No judicious physician could possibly regard this as a genuine case of Dilatation of the Heart.

Chap. VII., on the Diseases of the Valves, is, like most of its predecessors, defaced by many errors and unguarded assertions. Our own conviction is much at variance with Dr. F.'s statement, that "there are few practitioners possessing much experience in auscultation, who, in tolerably clear cases, will fail to point out the valve or orifice affected." This is a refinement of diagnostic discrimination which is rarely (is it ever?) attained; and fortunately the knowledge, if possessed, would not be of much practical value. Dr. F. talks also of distinguishing the second sound of the pulmonary or right semilunar valves from that of the aortic ones: truly, it must require an ear of surpassing delicacy to attain to such excellence in Auscultation. But passing over these matters, we would ask for information, does clinical observation warrant the remark that "the pulse is very peculiar, almost a diagnostic in disease of the Mitral Valve, being small, weak, intermittent, and singularly irregular?" We had supposed that this character of the pulse is a very frequent accompaniment of disease of the aortic valves; and indeed our author himself expressly admits that it is so, in the very next page.

In the part of his work now under consideration, Dr. F. has again done us the honour of quoting largely from the pages of the "Medico-Chirurgical Review;" but, this time, he frankly makes reference to its pages.

The rules, which are laid down for the treatment of Valvular Disease, are, it may be supposed, rather preventive than curative. Reverting to his favourite pathological and therapeutic views, he says :—

"We now know from accumulated experience, that Endocarditis is a fertile source of Valvular mischief; and that in that disease there are causes in operation which powerfully favour deposition on the Endocardium; viz., an inflammatory action of the membrane itself, and an excess of fibrine in the blood; to which I would ask permission to add, a peculiar abnormal change in the components of the blood itself. As I have already alluded to this subject, I need say no more at present, than that a combination of antiphlogistic treatment, with alkalies, will prevent all deposition. In all cases, the antiphlogistic remedies must be proportioned to the activity of the inflammatory symptoms; and mercury is also an indispensable adjunct, for we now believe that mercury, besides being antiphlogistic, has a tendency to reduce the excess of fibrine—to thin the blood; —but it will not relieve the chemical change, Alkali will alone do that according to my experience; and very glad shall I be if my medical brethren will fairly try it." 144.

The reasoning in the following passage we confess that we do not quite understand. Is there not a misprint somewhere? "The treatment of all Valvular Disease has been hitherto considered, excepting by a very few practitioners, without reference to the site—to the valve diseased; yet in obstruction of the Mitral Valve our practice ought to differ materially from that pursued in Aortic Valve Disease. Indeed the indications are almost diametrically opposed; for in the former the hypertrophic action of the right ventricle requires to be reduced as quickly and completely as possible; or else the congestion of the lungs, which is a first and immediate effect when combined with the powerful propulsion of the right ventricle, will give rise to a dangerous hæmoptysis, though itself an effort of nature to save life."—P. 147.

Having at considerable length discussed the diseases of the valves, Dr. F. devotes a chapter to the treatment of Aneurism of the Aorta, and then proceeds to the interesting subject of Palpitation and Functional Disorders. The occasional uncertainty of the diagnosis between these and the organic diseases of the heart is, in our author's opinion, not to be much regretted! "This circumstance," says he, "will at times prove a comfort to both practitioner and patient; as it should prevent the former from either giving or entertaining too gloomy an opinion while he awaits the results of treatment, and the latter can entertain more hope of recovery than if his medical adviser could decide that there is organic and incurable disease."

He very abruptly brings his remarks on this head to a close, and scarcely deigns to notice the treatment of functional cardiac diseases. To compensate for this unlooked-for deficiency, we are favoured with a long extract, running to nearly 20 pages, from the recent work of Mr. Sibson, of Nottingham, entitled "On the Changes induced in the situation and structure of the Internal Organs under varying circumstances of Health and Disease": it was reviewed in the last number of this Journal. This work Dr. F. mentions, in his Preface, as being "printed, though not yet published." How comes this? we received Mr. Sibson's work at least two months before Dr. Furnivall's. There must be a mistake somewhere, either on the part of the authors themselves, or their publishers. It is but fair to state that Dr. F. received full permission from Mr. Sibson to make any use that he pleased of his work.

From the preceding notice, the reader will be able to form an estimate for himself of the value of our author's production. Although exceedingly faulty in not a few respects, there are, it will be seen, some good points about it. Many of the therapeutic directions are judicious and valuable. The importance of alkalies in the treatment of various morbid conditions of the circulation is not sufficiently appreciated in the present day; and we think, therefore, that Dr. Furnivall has done good service to his readers by so strongly recommending their administration in acute Rheumatism, whether it be complicated with a cardiac affection, or not. We cannot too urgently advocate the expediency of examining the state of the urine in almost all diseases. Whenever it is scanty and deep-coloured, or when it deposits a lateritious sediment, alkaline medicines will invariably be useful, be the existing disease what it may; and certainly in none does the secretion usually exhibit these characters so conspicuously, as in acute rheumatism and in scarlet fever. But even when there is no obvious or visible change to be perceived, it is always right to test the quality of the urine with Litmus and Turmeric paper. Many an obstinate and most unyielding ailment has been relieved, by attending to the therapeutic indications afforded by this simple expedient.

With respect to the sedative—aconite—which Dr. Furnivall so strongly recommends in heart-diseases, we cannot speak from experience. That it is often truly useful in chronic rheumatism is amply attested by many writers: but that it possesses any *defibrinating* power upon the blood, seems very doubtful. Opium, in combination with magnesia, will generally soothe some of the most distressing symptoms of cardiac disease better than any other remedy of its class; especially if a mustard poultice or some other rubefacient be applied over the region of the heart, at the same time. But there is one sedative that is, we fear, little thought of, and far too little recommended by medical men, although it will often tranquillise the patient's sufferings more effectually than all the resources of pharmaceutic skill. How painful is it to witness the restlessness and irritability alike of mind and body, that are so generally present in chronic diseases of the heart! and how vain are often the physician's best attempts to afford relief! In no set of maladies are the effects of the close alliance and mutually reacting sympathy of mind and body so strikingly manifest. Every excitement of the one and every exertion of the other are almost inevitably followed by a greater or less disturbance of the current of blood through the chambers of the heart, and consequently by an increase in the amount of inward suffering. Hence the danger of any violent emotion, or any hasty effort; and hence the frequent occurrence of sudden death under circumstances too that are most truly lamentable. Surely then it is our duty to use and recommend every means to avert such a consequence, and to soothe, if we cannot heal, the sufferings of our patients; and where is the sweet influence that can so well subserve our wants, as the still small voice of that religious faith that teaches us "that we should patiently, and with thanksgiving, bear our heavenly Father's correction, whensoever by any manner of adversity it shall please His gracious goodness to visit us"?

